# Determinants of patient and health care services delays for tuberculosis diagnosis in Italy: a cross-sectional observational study

**DOI:** 10.1186/s12879-018-3609-4

**Published:** 2018-12-20

**Authors:** Anna Maria Peri, Davide Paolo Bernasconi, Nadia Galizzi, Alberto Matteelli, Luigi Codecasa, Vincenza Giorgio, Antonio Di Biagio, Fabio Franzetti, Antonella Cingolani, Andrea Gori, Giuseppe Lapadula

**Affiliations:** 1 Division of Infectious Diseases, “San Gerardo” Hospital, Via GB Pergolesi 33, Monza, Italy; 2 Infectious Diseases Unit, Fondazione IRCCS Ca’ Granda, Ospedale Maggiore Policlinico, University of Milan, Milan, Italy; 3 Centre of Biostatistics for Clinical Epidemiology, School of Medicine and Surgery, University of Milano-Bicocca, Milan, Italy; 4 “San Raffaele” Hospital, “Vita-Salute” University, Milan, Italy; 5 Department of Infectious and Tropical Diseases, WHO Collaborating Centre for TB/HIV and TB elimination, University of Brescia, Brescia, Italy; 6 Regional TB Reference Centre, “Villa Marelli” Institute, Niguarda Hospital, Milan, Italy; 7 “L. Fallacara” Hospital, Triggiano, BA Italy; 8 Infectious Diseases Department, IRCCS AOU San Martino-IST, Genova, Italy; 9 “L. Sacco” Hospital, University of Milan, Milan, Italy; 10 Policlinico “Gemelli”, “Sacro Cuore” Catholic University, Rome, Italy

**Keywords:** Tuberculosis, Diagnostic delay, Access to care

## Abstract

**Background:**

Prompt diagnosis of active tuberculosis (TB) has paramount importance to reduce TB morbidity and mortality and to prevent the spread of *Mycobacterium tuberculosis*. Few studies so far have assessed the diagnostic delay of TB and its risk factors in low-incidence countries.

**Methods:**

We present a cross-sectional multicentre observational study enrolling all consecutive patients diagnosed with TB in seven referral centres in Italy. Information on demographic and clinical characteristics, health-seeking trajectories and patients’ knowledge and awareness of TB were collected. Diagnostic delay was assessed as patient-related (time between symptoms onset and presentation to care) and healthcare-related (time between presentation to care and TB diagnosis). Factors associated with patient-related and healthcare-related delays in the highest tertile were explored using uni- and multivariate logistic regression analyses.

**Results:**

We enrolled 137 patients, between June 2011 and May 2012. The median diagnostic delay was 66 days (Interquartile Range [IQR] 31–146). Patient-related and healthcare-related delay were 14.5 days (IQR 0–54) and 31 days (IQR: 7.25–85), respectively. Using multivariable analysis, patients living in Italy for < 5 years were more likely to have longer patient-related delay (> 3 weeks) than those living in Italy for > 5 years (Odds Ratio [OR] 3.47; 95% Confidence Interval [CI] 1.09–11.01). The most common self-reported reasons to delay presentation to care were the mild nature of symptoms (82%) and a good self-perceived health (76%). About a quarter (26%) of patients had wrong beliefs and little knowledge of TB, although this was not associated with longer diagnostic delay. Regarding healthcare-related delay, multivariate analysis showed that extra-pulmonary TB (OR 4.3; 95% CI 1.4–13.8) and first contact with general practitioner (OR 5.1; 95% CI 1.8–14.5) were both independently associated with higher risk of healthcare-related delay > 10 weeks.

**Conclusions:**

In this study, TB was diagnosed with a remarkable delay, mainly attributable to the healthcare services. Delay was higher in patients with extra-pulmonary disease and in those first assessed by general practitioners. We suggest the need to improve knowledge and raise awareness about TB not only in the general population but also among medical providers. Furthermore, specific programs to improve access to care should be designed for recent immigrants, at significantly high risk of patient-related delay.

**Trial registration:**

The study protocol was registered under the US National Institute of Health ClinicalTrials.gov register, reference number: NCT01390987. Study start date: June 2011.

**Electronic supplementary material:**

The online version of this article (10.1186/s12879-018-3609-4) contains supplementary material, which is available to authorized users.

## Background

The delay in diagnosis of tuberculosis (TB) is one of the main factors contributing to the spread of *Mycobacterium tuberculosis* and preventing its elimination. Several studies reported a remarkable delay in TB diagnosis, often longer than 2 months. Both patients and healthcare-services can contribute to TB diagnostic delay, the former deferring their presentation to care and the latter missing the opportunity of a well-timed diagnosis. While most of the studies on this topic have been conducted in settings with limited resources and high TB incidence [1–11], substantial delays in TB diagnosis have been described also in high-resource settings [12–17]. A meta-analysis of the studies conducted between 1990 and 2008 showed that the mean diagnostic delay, including the proportion attributable to healthcare system, is not significantly different in constrained-resource and high-resource countries [18]. Determinants of TB diagnostic delay vary according to different studies and geographical contexts. Some studies reported that gender and age are associated with delayed TB diagnosis, with women and the elderly experiencing the highest delays [1, 4, 8, 11, 16, 17]. Social factors, such as unemployment, low income and low-grade education, have also been shown to influence time to TB diagnosis, particularly in low- and mid-resource settings [1, 7, 8, 11]. In addition, some studies from high-resource countries with low TB prevalence reported that TB diagnosis may require longer time in autochthonous patients than in immigrants [13, 15, 17]. In particular, in a study conducted in Norway, the delay attributable to healthcare services was significantly longer in patients born in Norway compared to patients born abroad. Of note, 86% of the 83 patients affected by TB included in the study were born abroad [13]. Similarly, an Italian survey from 2003 reported that a long delay attributable to healthcare services was significantly more frequent in Italians than in migrants while a long delay attributable to patients was significantly more frequent in recent migrants compared to Italians [15]. Also in UK, autochthonous patients had been demonstrated to have longer healthcare services delays than those born outside the UK [17].

Extra-pulmonary TB is often and universally reported as an important risk factor for diagnostic delay, because of its often non-specific signs and symptoms [14, 17]. This is true not only for high-incidence countries but also for low-incidence ones. Indeed, extra-pulmonary TB was associated with longer health care delay in Denmark, Norway and the UK [14, 15, 17].

With 4032 new cases diagnosed during the year 2016, incidence of TB in Italy has been estimated around 6.1 (5.3–7.1) per 100,000 population [19] with up to 50% of cases affecting migrants [20].

Country-specific assessment of TB diagnostic delay and identification of the determinants of such delay is essential to improve the access to care of patients with TB. Therefore, we performed a cross-sectional multicentre observational study, aimed at assessing patient- and healthcare-related delay in TB diagnosis in Italy and the risk factors associated with it.

## Methods

### Study design and setting

We conducted a cross-sectional multicentre observational study in seven referral centres for the diagnosis and treatment of TB in Italy, five in Northern Italy (“San Gerardo” Hospital, Monza; “Villa Marelli” Clinic, Milan; “Luigi Sacco” Hospital, Milan; “Spedali Civili” Hospital, Brescia, “San Martino” Hospital, Genova), one in Central Italy (“Policlinico Gemelli”, Rome) and one in Southern Italy (“Fallacara” Hospital, Bari).

### Participants

All consecutive adult patients (> 18 years old) diagnosed with TB between June 1st 2011 and May 30th 2012 were considered for enrolment. Patients with TB at any site (pulmonary and/or extra-pulmonary) were included. Patients whose clinical conditions interfered with their ability to comply with the study procedures or those who were unable to fill questionnaires because of lack of linguistic competence, as assessed by the investigators, were excluded.

The study protocol was approved by the Ethical Committees in all participant centres and was conducted according to Good Clinical Practice principles in agreement to the Declaration of Helsinki. All patients signed an informed consent before enrolment. The study protocol was registered under the US National Institute of Health ClinicalTrials.gov register, reference number: NCT01390987. Study start date: June 2011.

### Variables and data measurement

Upon enrolment, the investigator recorded patients demographic, epidemiological and clinical data. The following demographic and epidemiological variables were collected: gender, age, nationality, year of arrival in Italy, school degree, occupation, annual income and housing condition. Clinical variables included: site of TB, symptoms at presentation, dates and results of TB diagnostic procedures, presence of comorbidities and risk factors for mycobacterial infections (i.e., congenital or acquired immunosuppressive conditions including HIV, cirrhosis, neoplasms, diabetes, chronic renal failure, recent incarceration, alcohol or drug abuse), family history of TB, previous history or exposure to TB.

Patients were asked to report the date of symptoms onset and the date and type of all the contacts with a physician or a medical care facility since then. Symptoms at TB presentation were explored with a self-administered questionnaire and their frequency was graded on a semi-quantitative scale (“never”, “sometimes”, “often”, “very often”). Patients’ beliefs on social impact of the disease and their knowledge about TB were also explored through a questionnaire, whose items were derived from a previous South African study and adapted to the Italian setting [21]. In particular, knowledge about multidrug resistant TB and correlation between TB and HIV were not explored, considering the low incidence of these conditions in our country. Eventually, patients were also asked to report the reasons that, in their opinion, had delayed their access to medical observation and to give suggestions about how to accelerate the access to care.

### Bias

Results might be biased by the exclusion from enrolment of those patients not able to fill in the questionnaires because of linguistic barriers or particularly severe clinical conditions. Furthermore, recall bias should also be addressed, reflecting differences in the accuracy of the recollections retrieved by study participants.

### Study size

Due to the observational nature of the study the size of the study sample was not formally calculated based on statistical power analysis.

### Quantitative variables and statistical methods

Diagnostic delay was defined as the time between the onset of the symptoms and the date of the diagnosis of TB and further distinguished in patient-related and healthcare-related delay. Patient-related delay was defined as the time between the onset of symptoms of TB and the first time the patient sought medical care. Healthcare-related delay was defined as the time between the first presentation to care and the diagnosis of TB. Furthermore, treatment delay was defined as the time between diagnosis of TB and anti-TB treatment initiation.

Patients’ characteristics were summarized using numbers (percentages) for categorical variables and median (Interquartile Range [IQR]) for numerical variables. Uni- and multivariable logistic regression models were used to assess the association between high patient-related delay (defined as >2nd tertile of the delay distribution) and the following factors: gender, age, nationality, years lived in Italy, housing conditions, school degree, occupation, annual income, risk factors for mycobacterial infections, site of infection, symptoms, healthcare structure of first assessment, reasons reported by patients as responsible of delaying access to care, beliefs and knowledge about TB. All factors considered in the univariable models were also included in the multivariable model except for housing conditions, school degree and occupation, which were excluded due to sparse data. Similar models were run to explore possible associations between high healthcare-related delay (defined as >2nd tertile of distribution) and the same factors (with the exclusion of patients’ self-reported reasons for delaying access to care and their beliefs and knowledge about the disease) plus patient delay. Again, all factors considered in the univariable models were also included in the multivariable model, except for housing conditions, school degree and occupation. In order to exclude that the choice of a specific cut-off could have influenced the results, quantile regression was performed, considering healthcare- and patient-related delays as continuous variables (the same set of covariates included in the corresponding logistic models was used).

All presented P are two-sided with a *P* < 0.05 indicating conventional statistical significance. All analyses were performed using R software version 3.3.2.

## Results

Among 163 patients evaluated for the study, 137 were enrolled. Reasons of exclusion from enrolment were: inability to sign the informed consent (8 patients), language barrier (*n* = 5), patient declined to participate to the study (*n* = 8), loss to follow up or death after the first visit (*n* = 4 and 1, respectively). Six patients returned blank questionnaires, thus information on symptoms, healthcare seeking pathway, knowledge and awareness of TB was available for 131/137 patients.

### Patients characteristics

The median age at patient enrolment was 40 (IQR: 29–56) years. Most participants were male (87/137, 63%) and foreign-born (95/137, 69%). About half of the patients (70/137) reported to have no income or to earn < 10,000€ per year and 7% (10/137) were unemployed. Forty-three percent (57/132) of the patients attended school for < 8 years. Exclusive extra-pulmonary TB involvement was diagnosed in about a third of the study participants (38/137). Diagnosis of TB was confirmed by positive cultures for *M.tuberculosis* in 82/137 cases (59.8%). Among the remaining patients, diagnosis was based on the presence of acid-fast bacilli at microscopy in 13/137 cases (9.5%), on positive molecular tests (*M.tuberculosis*-PCR) in 10/137 (7.3%), and on histology in 12/137 cases (8.8%). In the last 20/137 (14.6%) patients, a clinical diagnosis of TB was established defined as signs, symptoms and clinical history consistent with TB in association to clinical response to empirical treatment with anti-TB medication within 2 months of treatment initiation.

Complete patient characteristics are listed in Table 1.

**Table 1:** Characteristics of the 137 patients diagnosed with tuberculosis enrolled in the study

Characteristic	*N* = 137
Female gender	50 (36.5)
Age (median [IQR])	40 [29, 56]
Nationality	
Italian	42 (30.7)
African	31 (22.6)
South-American	17 (12.4)
Asian	27 (19.7)
Eastern European	20 (14.6)
Years lived in Italy (*N* = 95)	
<2	11 (11.6)
2–5	20 (21.0)
>5	49 (51.6)
Housing conditions	
Living with others	46 (33.6)
Living with family	71 (51.8)
Living alone	20 (14.6)
Have children	84 (61.3)
School degree	
Primary school degree	26 (19.0)
Secondary school degree	31 (22.6)
High school diploma	65 (47.4)
University degree	10 (7.3)
Occupation	
Unemployed	10 (7.3)
Employed/Retired	117 (85.4)
Student	10 (7.3)
Annual income	
No income	28 (20.4)
<10,000 €	42 (30.7)
10,000–30,000 € per year	42 (30.7)
>30,000 per year	6 (4.4)
HIV and other comorbidities	
HIV infection	10 (7.3)
Immunosuppressive therapies	5 (3.6)
Haematological diseases	2 (1.5)
Chronic kidney disease	2 (1.5)
Neoplasms	6 (4.4)
Cirrhosis	4 (2.9)
Diabetes	10 (7.3)
Social conditions	
Alcohol abuse	3 (2.2)
Drug abuse	2 (1.5)
Homelessness	3 (2.2)
Past imprisonment	5 (3.6)
History of potential exposure	
Family history	7 (5.1)
Professional exposure	3 (2.2)
Contact with TB patient	8 (5.8)
Site of involvement	
Extra-pulmonary	38 (27.7)
Pulmonary	78 (56.9)
Pulmonary and extra-pulmonary	20 (14.6)
Case Definition	
New Case	117 (85.4)
Relapse or previous failure	14 (10.2)
Diagnosis	
Culture	82 (59.8)
Microscopy	13 (9.5)
Molecular test	10 (7.3)
Histology	12 (8.8)
Clinical	20 (14.6)
Respiratory symptoms^a^	88 (67.2)
Healthcare structure of first assessment^a^	
General practitioner	55 (42.0)
Specialist consultation	25 (19.2)
Emergency Department	39 (29.8)
Other	11 (8.5)
Reasons for delay^a,b^	
Underestimation of symptoms	98 (74.8)
Fear of consequences	54 (41.2)
Barriers to healthcare access	49 (37.4)
Good TB knowledge^a^	80 (61.1)
Uncorrect beliefs about TB^a^	76 (58)

All results are presented as number of occurrences with percentages in brackets, unless otherwise specified

^a^Percentages calculated on the total of 131 patients who returned a filled questionnaire

Abbreviations: *HIV* human immunodeficiency virus, *IQR* inter-quartile range, *N/A* not applicable, *TB* tuberculosis

^b^“Underestimation of symptoms” includes the following self-reported reasons in the questionnaire: “symptoms did not seem to be important”, “overall I felt well”;

“Fear of consequences” includes the following self-reported reasons: “I was afraid of a positive result”, “I was afraid I would be reported to the authorities”, “I was afraid to lose my job”, “I was afraid to be rejected by my friends”, “My friends discouraged me”

“Barriers to healthcare access” includes the following self-reported reasons: “I did not have time to go to the doctor”, “I did not know where to go”, “I did not have money”

### Diagnostic delay

The median diagnostic delay was 66 days (IQR 31–146) since the onset of the symptoms. In particular, the median delay attributable to patients was 14.5 days (IQR 0–54), whilst the median healthcare-related delay was 31 days (IQR 7.25–85). Antimycobacterial treatment was initiated the same day of the diagnosis or the day after in most of patients (median therapeutic delay: 0 days, IQR: 0–3). Diagnostic delay was significantly higher among subjects with extra-pulmonary than among those with pulmonary TB. This difference was mainly driven by a significant difference in healthcare-related delay (54 days [IQR 24–122] versus 17 days [IQR 3–63] among subjects with extra-pulmonary versus pulmonary TB, respectively; Mann-Whitney *p* < 0.001). Although also patient-related delay was higher among those with extra-pulmonary than among those with pulmonary TB, such difference did not reach statistical significance (27 days (IQR 0–58) versus 10 days (IQR 1–49); Mann-Whitney *p* = 0.443).

Notably, the median diagnostic delay did not statistically differ between Italian and foreign-born patients (68 days [IQR: 40–157] versus 65 days [IQR: 31–136], respectively; Mann-Whitney *p* = 0.579), nor it differed after stratification for site of infection. In detail, diagnostic delay of patients with pulmonary TB was, in median, 62 days (IQR 28–139) among Italians and 45 days (IQR 20–103) among foreign-born patients (Mann-Whitney *p* = 0.278). The median diagnostic delay of patients with extra-pulmonary TB was 94 days (IQR 54–276) among Italians versus 91 days (IQR 49–156) among foreign-born patients (Mann-Whitney *p* = 0.659).

There was no evidence of any statistically significant correlation between patient-related and healthcare-related delay (Spearman correlation index 0.17).

**Fig. 1:**
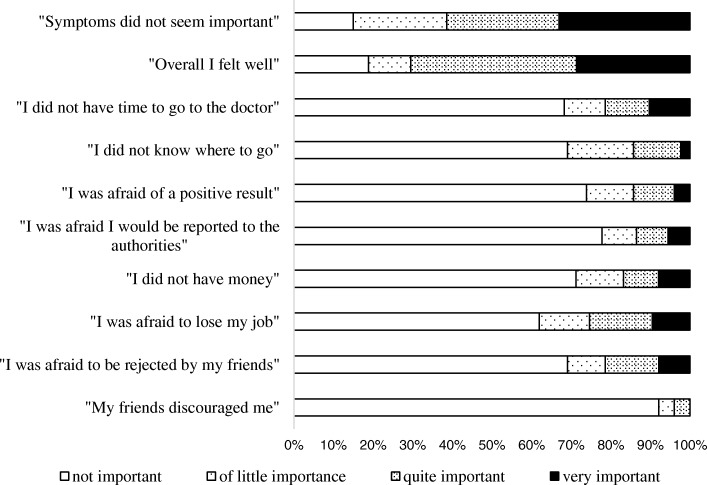
Self-reported reasons for delayed access to care

The most commonly reported reasons that led patients to procrastinate their presentation to care were the mild nature of the initial symptoms (108/131, 82%) and a good self-perceived health (99/131, 76%). A lower, but significant, proportion of patients, ranging from 17 to 32%, reported fear of the consequences of a diagnosis, fear to be reported to the authorities, lack of knowledge of the health system, time and economical constraints as additional barriers to the access to care. Figure 1 shows the complete results of the questionnaire aimed at assessing the reasons of delayed access to medical observation.

### Knowledge and beliefs about tuberculosis

Almost two thirds of the patients (80/131, 61%) had a good knowledge about TB route of transmission, epidemiology and prognosis. In particular, 92% (120/131) of the patients were aware that TB is a communicable disease and is transmitted from person to person by direct contact, although only 82% (108/131) knew it spreads through airborne transmission. Whilst 6% (8/131) thought that TB is an untreatable disease, 94% (123/131) reported it could be cured with antibiotics. Most patients (127/131, 97%) were well aware of the risk of serious health consequences for those not taking treatment, but 18% (23/131) denied that untreated TB could lead to death. Of note, 15% (20/131) believed that TB only affects immigrants or people with pre-existing health problems.

A considerable number of study participants (28/131, 21%) had self-blame feelings, attributing the acquisition of TB to their own misbehaviour, while 27% (36/131) of the study population believed that they got TB due to wrong behaviours of other people. Eventually, 26% (34/131) of the patients reported that people affected by TB should be secluded from the society.

### Risk factors for patient-related delay

Table 2 shows uni- and multivariable analyses investigating factors associated with a patient-related delay in the highest tertile (> 3 weeks). Using univariate analysis, no significant association was found between patient-related delay and age, gender, school degree, occupation, annual income, housing condition and nationality. Nonetheless, foreign-born patients living in Italy for < 5 years were more likely to present late to medical observation than Italians or foreign-born patients who had lived in Italy for ≥5 years (Odds Ratio [OR] 2.45; 95% Confidence Interval [CI] 1.03–5.87; *p* = 0.043). This result was confirmed in the multivariate analysis, after adjusting for possible confounders (Adjusted OR 3.47; 95% CI 1.09–11.01; *p* = 0.035). Moreover, using multivariable analysis, symptoms underestimation was also marginally associated with a higher patient-related delay (Adjusted OR 3.43; 95% CI 0.98–12.04; *p* = 0.055). Wrong knowledge and beliefs on TB, on the contrary, were not significantly associated with a delayed presentation to care.

**Table 2:** Univariate and multivariate regression analysis assessing risk factors for patient diagnostic delay > 3 weeks

Factors	Univariate analysis	Multivariate analysis
	OR (95% CI)	OR (95% CI)
Age (years)	*1 (0.98; 1.02)*	*1.03 (1; 1.06)*
Female gender	1.52 (0.74; 3.13)	1.72 (0.68; 4.39)
Nationality		
Italian	1	1
African	1.66 (0.61;4.50)	2.91 (0.62; 13.60)
South-American	1.25 (0.39;4.01)	1.54 (0.33; 7.15)
Asian	1.79 (0.65;4.90)	1.48 (0.33; 6.56)
Eastern European	1.98 (0.65;6.04)	3.19 (0.64; 15.76)
Living in Italy for < 5 years	*2.46 (1.03;5.87)*	*3.47 (1.09; 11.01)*
Annual income		
≥10,000 €	1	1
<10,000 €	0.930 (0.43;2)	1.21 (0.47; 3.14)
No source of income	1.89 (0.64;5.58)	2.84 (0.53; 15.24)
Housing conditions		
Living with others	1	
Living with family	1.14 (0.53;2.47)	
Living alone	0.47 (0.14;1.54)	
School degree		
University degree	1	
Primary/secondary school degree	1.50 (0.38;5.88)	
High school diploma	1.00 (0.26;3.92)	
Occupation		
Unemployed	1	
Employed/Retired	0.82 (0.20;3.44)	
Student	0.50 (0.07;3.55)	
Comorbidities	0.79 (0.34;1.80)	0.95 (0.33; 2.70)
Social conditions	1.61 (0.41;6.30)	1.50 (0.27; 8.25)
History of potential exposure	0.68 (0.30;1.54)	0.61 (0.22; 1.71)
Extra-pulmonary TB	1.36 (0.63;2.94)	1.43 (0.51; 4.03)
Absence of respiratory symptoms^a^	0.96 (0.46;2.03)	1.38 (0.53; 3.61)^a^
First assessment by General Practitioner	0.93 (0.45;1.88)	0.63 (0.24; 1.64)
Reasons for delay^b^		
Underestimation of symptoms	1.47 (0.65;3.34)	3.43 (0.98; 12.04)
Fear of consequences	0.87 (0.43;1.77)	1.01 (0.34; 2.98)
Barriers to healthcare access	1.02 (0.50;2.10)	1.35 (0.49; 3.74)
Bad TB knowledge	1.17 (0.57;2.40)	1.23 (0.48; 3.19)
Uncorrect beliefs about TB	0.78 (0.38;1.57)	0.59 (0.23; 1.51)

Statistically significant results are shown in italics

^a^assessed in a separate multivariable model not including extra-pulmonary TB

Abbreviations: *OR* Odd Ratio, *CI* Confidence Interval, *TB* Tuberculosis

^b^For details of self-reported reasons for delay see Table 1

Sensitivity analysis using quantile regression (full results shown in Additional file 1: Table S1) yielded to only slightly different results, suggesting older age (difference of the medians 0.05; 95% CI 0.03–0.09; *p* = 0.019) to be a possible additional factor associated with longer patient-related delay.

### Risk factors for healthcare-related delay

Table 3 shows the results of uni- and multivariable analyses assessing factors associated with healthcare-related delay in the highest tertile (> 10 weeks). Using univariate analysis, the following factors were significantly associated with a healthcare-related delay: absence of respiratory symptoms (OR 2.57; 95% CI 1.19–5.55; *p* = 0.016), first assessment performed by the general practitioner (OR 5.95; 95% CI 2.66–13.31; *p* < 0.001) and extra-pulmonary TB (OR 4.09; 95% CI 1.82–9.22; *p* = 0.001). Using multivariate analysis, first assessment by the general practitioner was confirmed to be independently associated with a higher risk of diagnostic delay attributable to the healthcare system (Adjusted OR 5.09; 95% CI 1.78–14.54; *p* = 0.002). Extra-pulmonary involvement (Adjusted OR 4.34; 95% CI 1.36–13.81; *p* = 0.013) and absence of respiratory symptoms (OR 3.29; 95% CI 1.18–9.23; *p* = 0.023) were also confirmed to be two additional independent predictors of healthcare-related delay, although these two variables were explored in two separated multivariate models, in order to avoid multicollinearity.

**Table 3:** Univariate and multivariate regression analysis assessing risk factors for healthcare system diagnostic delay > 10 weeks

Factors	Univariate analysis	Multivariate analysis
	OR (95% CI)	OR (95% CI)
Age (years)	1.00 (0.98;1.02)	1 (0.96; 1.03)
Female gender	1.60 (0.75;3.38)	1.90 (0.65; 5.55)
Nationality		
Italian	1	1
African	1.43 (0.52;3.93)	0.63 (0.11; 3.57)
South-American	0.45 (0.11;1.83)	0.20 (0.03; 1.35)
Asian	1.43 (0.52;3.93)	1.09 (0.23; 5.12)
Eastern European	0.55 (0.15;2.00)	0.58 (0.10; 3.38)
Living in Italy for < 5 years	1.09 (0.45;2.64)	1.00 (0.28; 3.65)
Annual income		
≥ 10,000 €	1	1
< 10,000 €	1.99 (0.89;4.45)	1.78 (0.60; 5.27)
No source of income	1.30 (0.43;3.97)	1.74 (0.39; 7.73)
Housing conditions		
Living with others	1	
Living with family	1.14 (0.51;2.55)	
Living alone	0.38 (0.09;1.51)	
School degree		
University degree	1	
Primary/secondary school degree	1.90 (0.37;9.82)	
High school diploma	2.10 (0.41;10.79)	
Occupation		
Unemployed	1	
Employed/Retired	3.55 (0.42;29.88)	
Student	3.50 (0.28;43.16)	
Comorbidities	0.57 (0.22;1.45)	0.58 (0.17; 2.04)
Social conditions	0.24 (0.03;2.02)	0.70 (0.07; 7.41)
History of potential exposure	0.50 (0.20;1.28)	0.67 (0.20; 2.27)
Extra-pulmonary TB	*4.09 (1.82;9.22)*	*4.34 (1.36; 13.81)*
Absence of respiratory symptoms	*2.57 (1.19;5.55)*	*3.29 (1.17; 9.23)* ^*a*^
First assessment by the General Practitioner	*5.95 (2.66;13.31)*	*5.09 (1.78; 14.54)*
Patient delay (per week longer)	1.00 (0.99;1.02)	0.99 (0.97; 1.01)

Statistically significant results are shown in italics

^a^assessed in a separate multivariable model not including extra-pulmonary TB

Abbreviations: *OR* Odd Ratio, *CI* Confidence Interval, *TB* Tuberculosis

No association was found between healthcare-related delay and other explored covariates, including patient nationality, demographic and social characteristics.

Using quantile regression (Additional file 1: Table S2), first assessment by General Practitioner (difference of the medians 6.45; 95% CI 4.23–9.51; *p* < 0.001), extra-pulmonary involvement (difference of the medians 5.51; 95% CI 2.25–10.52; *p* < 0.001) and absence of respiratory symptoms (difference of the medians 2.53; 95% CI 1.08–10.40; *p* = 0.001) were confirmed to be associated with longer healthcare-related delay.

### Health seeking pathways

**Fig. 2:**
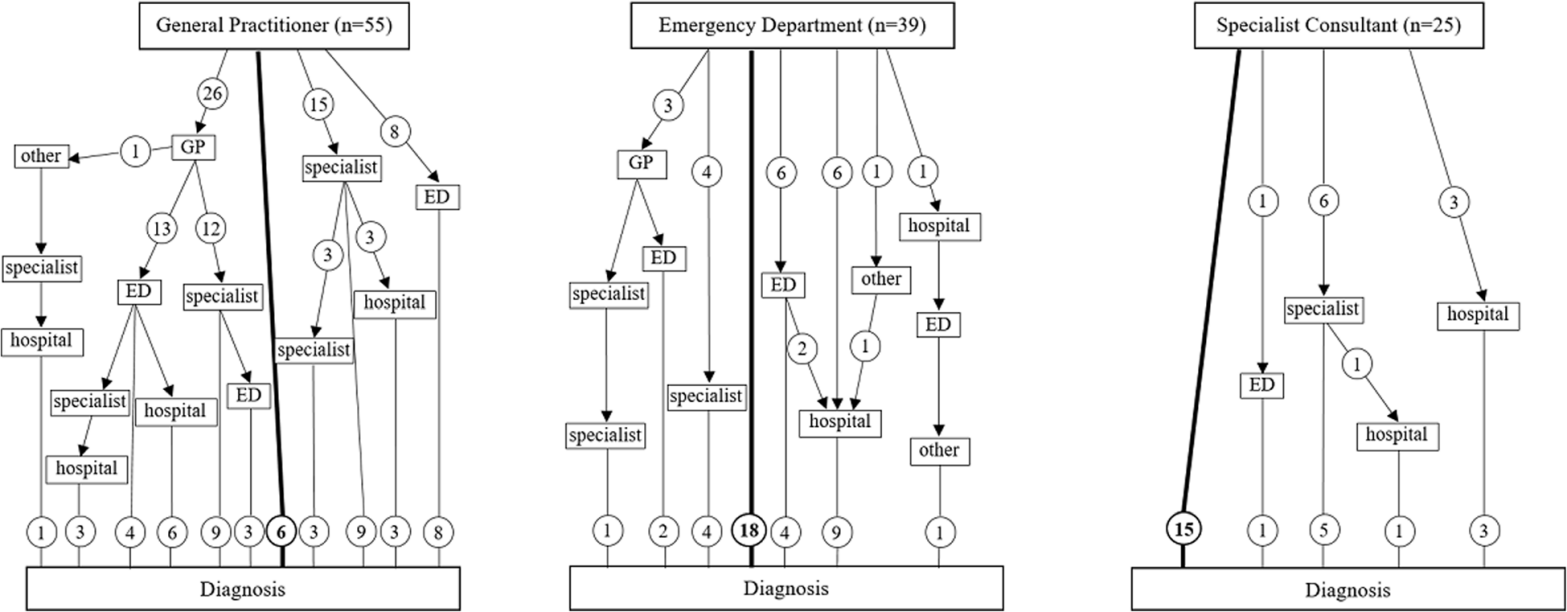
Healthcare seeking pathways of enrolled patients according to the first medical provider consulted. Numbers in circles refer to patients assessed by different medical providers before diagnosis of tuberculosis. Abbreviations: ED, emergency department; GP, general practitioner

Overall, the median number of medical consultation or contacts with an healthcare facility before diagnosis was 2 (IQR 1–3). Figure 2 shows the pathways followed by the patients, from the first time they presented to a healthcare provider up to the diagnosis. The health-seeking pathways are distinguished according to the first healthcare provider the patients consulted (general practitioner, emergency department or specialist consultant). Patients that initially sought medical care from their general practitioner consulted an average of 2.8 healthcare providers before the diagnosis, patients evaluated in a hospital-based Emergency Department had a mean of 1.8 consultations before receiving the correct diagnosis, whereas patients that were first evaluated by a specialist consultant in outpatient clinics were diagnosed after a mean of 1.4 consultations. Suspect of tuberculosis was raised after the first contact in 46% (18/39) of patients presenting to an emergency departments, in 60% (15/25) of those attending a specialist consultation and in 11% (6/55) of the patients evaluated by the general practitioner.

## Discussion

Herein we report a cross-sectional observational study assessing the diagnostic delay in the diagnosis of tuberculosis in seven TB referral centres in Italy. A considerable latency between symptom onset, clinical presentation and TB diagnosis was observed. In most of the patients, the diagnostic delay was longer than 2 months and about a quarter of the population was diagnosed after > 5 months since the onset of the symptoms. Our results are consistent with those from previous studies [5, 8, 12–18] and confirm that unacceptable diagnostic delays still exist, in high-resource as well as in resource-constrained settings. In our study, the diagnostic delay attributable to healthcare services was about two-times longer than that attributable to patients (in median, 31 versus 14 days, respectively). Although this finding is similar to a previous regional survey conducted in Italy [15], several other studies conducted in high-resource settings showed patients and healthcare service to be equally responsible for the overall diagnostic delay of TB [12–14, 16, 22, 23]. Possible explanations for this apparent discrepancy might depend on the proportion of elderly and foreign-born patients in the general population and on differences between healthcare systems, in particular in their arrangements for migrants, translating into variable access to care.

We found that native and non-native patients had similar time of presentation to care. Nonetheless, when we distinguished patients according to the time of their first arrival in Italy, those who lived in Italy for < 5 years had considerable higher risk of late presentation to care than long-term and permanent residents. This suggests that recently immigrated subjects experience significant difficulties in accessing to Health Services, possibly because of bureaucratic, legal and economical barriers, which were, indeed, reported by 12–18% of the patients. By converse, foreign-born patients who had settled in Italy for ≥5 years behaved similarly to native Italians. Given the fact that recent immigrants are also those with the highest risk of TB reactivation/reinfection, specific screening program, targeted information campaigns and social inclusion programs could be beneficial for these patients. Symptoms underestimation was the most common reason leading patients not to seek early medical care and, in multivariate model, it was associated with a > 3-fold increased risk of high patient-related delay. In this respect, promoting an adequate knowledge about TB risk and its clinical presentation is advisable.

We did not find any significant association between other socioeconomic determinants (such as level of education, employment status, annual income or family condition) and delayed presentation to care or health care-related delay. Despite the fact that the association between social disadvantage and TB has been well documented, this finding is not completely surprising because no single measure of socioeconomic status can capture all the aspects of social vulnerabilities and health inequalities that determine delayed diagnosis.

Healthcare related delay was, in our study, the main contributor to total TB diagnostic delay. Consistently with other studies [13, 14, 16, 17], extra-pulmonary TB involvement and lack of respiratory symptoms were associated with longer diagnostic delays. Unspecific symptoms, unusual clinical presentations and difficulties in obtaining specimens for the diagnosis are all likely explanation for this finding and reflect the challenges faced by clinicians during TB diagnosis. It is unrealistic to believe that, particularly in low-incidence countries, all TB can get to a prompt and rapid diagnosis and, from an epidemiological point of view, delays in extra-pulmonary TB diagnosis can look less important than delaying the diagnosis of other communicable forms of TB. Nonetheless, extra-pulmonary TB is often clinically severe and life-threatening and diagnostic delays can be associated with disease progression and a bad prognosis. Before a definite diagnosis is determined, a certain index of suspicion for TB disease should be maintained in patients with compatible symptoms and/or epidemiological risk factors, even when other diagnoses are deemed more likely.

Health-seeking pathways had high influence on diagnostic delay, with a major role played by the first health care provider consulted by patients. Patients initially assessed by the general practitioner experienced longer healthcare-related delay and needed a higher number of contacts with the healthcare system before diagnosis, than those who had direct access to the hospital or consulted a specialist. Various reasons may explain this difference. First, Italy is a low-incidence country and TB rates have steadily reduced in the last 50 years. In such setting, the ability of general practitioner to recognize and diagnose a disease that they rarely see is likely to have reduced over time. As a matter of fact, TB diagnostic delay in healthcare structures has been previously demonstrated to be inversely proportional to the number of cases annually diagnosed by the health-care structures themselves [24]. It is not surprising, therefore, that specialist physicians play an important role in case detection, nowadays. Nonetheless, more than 40% of the patients, in our study, referred to the general practitioner, in the first instance. Informative campaigns and continuous education programs on TB for general practitioners are therefore highly warranted and likely to have an impact on diagnostic delay. Another possible explanation for the health-seeking pathway impact on healthcare-related delay is that symptoms of patients presenting to the emergency department could be more severe or specific than those of patients who contacted their general practitioner, thus facilitating a more prompt diagnosis. Lastly, patients presenting to the emergency department or specialist consultation could also be those more likely to be perceived at risk for TB, because undocumented migrants and other marginalized patients could have easy and free access to hospital care and to TB facilities, but not to primary care.

Results from the present survey add new and important information about TB diagnostic delay in a low-incidence country. Some limitations of the study should be, however, acknowledged. In particular, the small sample size (about 10% of the TB cases notified every year in Italy), the exclusion from enrolment of patients due to language barriers and inability to sign informed consent and the heterogeneous geographical distribution of the TB referral centres included in the study may limit its generalizability. Nonetheless, it should be noted that the preponderance of centres located in large urban areas of Northern Italy included in our study reflects the higher burden of TB in these areas. Moreover, two important reference centres, one in Central and one in Southern Italy, were also involved. Another limitation of the study is the lack of previous validation of the questionnaire on knowledge and beliefs of the patients, which was indeed derived and adapted from a questionnaire previously validated in a different context [21], but not further validated on our population. On the other hand, despite the previously discussed recall bias, the use of specifically designed questionnaires is an invaluable strength of this research, because it allowed to collect precise data on patients’ socio-economic conditions, path of access to the healthcare services and patients’ beliefs and opinions. Finally, consensus about what duration between onset of TB symptoms and diagnosis is considered as delay is lacking in the literature and to this extent quantiles were used in our analysis. Nonetheless, the principle “the later, the worse” is certainly reasonable at least for highly contagious forms of TB.

## Conclusions

In conclusion, our study showed that TB diagnostic delay is common. Recently immigrated patients are those most at risk for a delayed presentation to care, whereas those with extra-pulmonary disease have lower chances of being diagnosed early after presentation for care. In addition, patient evaluated, in the first instance, by primary care physicians are more likely to delay the diagnosis and to need repeat consultations before TB is suspected. Since delaying TB diagnosis can have severe implications for patients health as well as for TB transmission, informative campaigns for patients and continuous education programs for physicians are highly warranted.

## Additional file

Additional file 1: **Table S1.** Results from quantile regression on patient-related delay. **Table S2** Results from quantile regression on healthcare-related delay. (DOCX 17 kb)

## Abbreviations

TB: Tuberculosis
