# Supplementary material for: Determinants of patient and health care services delays for tuberculosis diagnosis in Italy: a cross-sectional observational study
Source: BMC Infect Dis. 2018 Dec 20;18:690. doi: 10.1186/s12879-018-3609-4 (PMC6302482; doi:10.1186/s12879-018-3609-4)
Supplement: Supplementary file 1 — Table S1. Results from quantile regression on patient-related delay. Table S2 Results from quantile regression on healthcare-related delay. (DOCX 17 kb) [file 12879_2018_3609_MOESM1_ESM.docx]

**Supplementary table 1**. Results from quantile regression on patient-related delay.

| **Factors** | Quantile regression  **Difference of the medians (95% CI)** |
| --- | --- |
| Age (per year) | *0.05 (0.03; 0.09)* |
| Female gender | 0.46 (-0.30; 3.08) |
| Nationality |  |
| Italian | 1 |
| African | 0.52 (-0.44; 3.59) |
| South-American | *0.94 (0.03; 3.11)* |
| Asian | 0.41 (-0.81; 5.78) |
| Eastern European | *1.76 (0.54; 6.84)* |
| Foreign born patients in Italy for <5 years | *2.68 (0.81; 5.41)* |
| Annual income |  |
| ≥ 10000 € | 1 |
| < 10000 € | 0.19 (-1.47; 1.32) |
| No source of income | 2.31 (-1.26; 4.73) |
| Comorbidities | -0.04 (-2.18; 0.82) |
| Social conditions | 2.26 (-1.49; 5.46) |
| History of potential exposure | -1.44 (-1.88; 0.11) |
| Extra-pulmonary TB | 0.02 (-1.17; 1.92) |
| Absence of respiratory symptoms ^a^ | -0.26 (-1.78; 1.03) |
| First assessment by the General Practitioner | -0.14 (-2.30; 1.03) |
| Reasons for delay |  |
| Underestimation of symptoms | *2.56 (0.44; 4.97)* |
| Fear of consequences | -0.26 (-2.51; 1.07) |
| Barriers to access health care system | 0.48 (-0.94; 2.70) |
| Bad knowledge of TB (ref. good knowledge) | 0.99 (-1.34; 2.05) |
| Wrong perception of TB (ref. right perception) | -0.30 (-2.70; 1.34) |

Statistically significant results are shown in italics.

^a^ assessed in a separate multivariable model not including extra-pulmonary TB

Abbreviations: CI, Confidence Interval; TB, Tuberculosis

**Supplementary table 2**. Results from quantile regression on healthcare-related delay.

| **Factors** | Quantile regression  **Difference of the medians (95% CI)** |
| --- | --- |
| Age (years) | *0.049 (0.03; 0.13)* |
| Female gender | -0.04 (-2.17; 3.41) |
| Nationality |  |
| Italian | 1 |
| African | 0.85 (-3.70; 5.43) |
| South-American | -1.79 (-3.88; 2.00) |
| Asian | 2.24 (-0.48; 3.29) |
| Eastern European | 0.69 (-1.48; 3.29) |
| Foreign born patients in Italy for <5 years | 0.91 (-1.80; 3.46) |
| Annual income |  |
| ≥ 10000 € | 1 |
| < 10000 € | 2.74 (-0.32; 3.61) |
| No source of income | -0.18 (-5.60; 3.84) |
| Comorbidities | -0.44 (-2.74; 1.39) |
| Social conditions | *-2.63 (-4.38; -1.66)* |
| History of potential exposure | 0.74 (-1.56; 3.58) |
| Extra-pulmonary TB | *5.51 (2.25; 10.52)* |
| Absence of respiratory symptoms ^a^ | *2.53 (1.08; 10.40)* |
| First assessment by the General Practitioner | *6.45 (4.23; 9.51)* |
| Patient delay (per week longer) | -0.08 (-0.10; 0.05) |

Statistically significant results are shown in italics.

^a^ assessed in a separate multivariable model not including extra-pulmonary TB

Abbreviations: CI, Confidence Interval; TB, Tuberculosis
